# Cardiomyocyte-specific deletion of β-catenin protects mouse hearts from ventricular arrhythmias after myocardial infarction

**DOI:** 10.1038/s41598-021-97176-9

**Published:** 2021-09-06

**Authors:** Jerry Wang, Ying Xia, Aizhu Lu, Hongwei Wang, Darryl R. Davis, Peter Liu, Rob S. Beanlands, Wenbin Liang

**Affiliations:** 1 University of Ottawa Heart Institute, 40 Ruskin St, Ottawa, ON K1Y 4W7 Canada; 2 Department of Cellular and Molecular Medicine, University of Ottawa, Ottawa, ON Canada

**Keywords:** Cardiology, Cardiovascular biology, Arrhythmias, Heart failure

## Abstract

Wnt/β-catenin signaling is activated in the heart after myocardial infarction (MI). This study aims to investigate if β-catenin deletion affects post-MI ion channel gene alterations and ventricular tachycardias (VT). MI was induced by permanent ligation of left anterior descending artery in wild-type (WT) and cardiomyocyte-specific β-catenin knockout (KO) mice. KO mice showed reduced susceptibility to VT (18% vs. 77% in WT) at 8 weeks after MI, associated with reduced scar size and attenuated chamber dilation. qPCR analyses of both myocardial tissues and purified cardiomyocytes demonstrated upregulation of Wnt pathway genes in border and infarct regions after MI, including Wnt ligands (such as *Wnt4*) and receptors (such as *Fzd1* and *Fzd2*). At 1 week after MI, cardiac sodium channel gene (*Scn5a)* transcript was reduced in WT but not in KO hearts, consistent with previous studies showing *Scn5a* inhibition by Wnt/β-catenin signaling. At 8 weeks after MI when Wnt genes have declined, *Scn5a* returned to near sham levels and K^+^ channel gene downregulations were not different between WT and KO mice. This study demonstrated that VT susceptibility in the chronic phase after MI is reduced in mice with cardiomyocyte-specific β-catenin deletion primarily through attenuated structural remodeling, but not ion channel gene alterations.

## Introduction

Each year, ventricular tachyarrhythmia-induced sudden cardiac death (SCD) claims the lives of millions of people worldwide, including ~ 370,000 Americans^1^ and ~ 700,000 European people^2^. Ischemic heart disease (myocardial infarction, MI) is the most common underlying disorder and accounts for 65–80% of the SCD cases^3^. In post-MI hearts, ventricular tachyarrhythmias (including tachycardia and fibrillation, VT/VF)^4,5^ result from both structural remodeling of the injured heart, i.e. fibrosis and scar formation in the infarcted region, and alterations in expression of ion channels/transporters especially in the border zone of the infarct^6–8^, such as reduced Na_v_1.5^9^ and Cx43^10^ as well as re-distribution of Cx43 (less in intercalated disk, but more in lateral surface of myocytes)^10^. The scar tissues are often interspersed with bundles of alive cardiomyocytes, which form “reentry circuit” together with border zone myocardium that has reduced and heterogenous conduction velocity providing the substrate for VT/VF^6^. The alterations in ion channels in alive myocardium also increases their automaticity or triggered activity^11^ that can initiate VT/VF.

The Wnt signaling is critical for embryonic cardiogenesis^12^ and we have recently shown that it regulates the differentiation of embryonic stem cells into different subtypes of cardiomyocytes^13^. In the canonical Wnt/β-catenin pathway, the binding of Wnt ligands to their plasma membrane receptors leads to the cytoplasmic accumulation of β-catenin, followed by its translocation into the nucleus and activation or inhibition of the transcription of target genes. In healthy adult hearts, Wnt/β-catenin signaling has a low activity but recent studies have suggested that its activity is increased in the myocardium in both human ischemic heart failure^14^ and animal models of myocardial infarction^15^ and heart failure^16^, as well as in murine models of cardiac hypertrophy induced by pressure overload or angiotensin II^17–20^. However, one study reported that increased Wnt/β-catenin signaling was only found in the myocardium of post-MI pigs with hypercholesterolemia, but not in post-MI pigs with normal cholesterol levels^21^. Recent studies, including those from our group^22–24^, have demonstrated the capability of Wnt/β-catenin signaling to regulate the expression of cardiac sodium channel gene *Scn5a* (encoding Na_v_1.5)^22–28^. However, it remains unknown if Wnt/β-catenin signaling plays a role in the post-MI alterations in cardiac ion channel genes.

On the other hand, previous studies have not reached a consensus regarding the role of Wnt/β-catenin signaling in the structural remodeling of the post-MI hearts. Several studies have suggested that the Wnt/β-catenin signaling is cardioprotective and reduces scar size. In post-MI rats, viral expression of a constitutively active form of β-catenin in the border zone reduced infarct size, which is associated with reduced apoptosis and increased cell proliferation in both cardiomyocytes and fibroblasts^29^. In mice with deletion of Lrp5, the co-receptor required for Wnt/β-catenin pathway activation, the infarct size after MI was larger than in wild-type mice^21^. In mice with fibroblast-specific deletion of β-catenin, ischemia/reperfusion injury induced accelerated LV dilation and pump dysfunction^15^. By contrast, other studies have suggested a detrimental role of Wnt/β-catenin signaling. In post-MI mice, enhancement of the Wnt/β-catenin signaling in myocardium via Wnt3a protein injection accentuated cardiac dysfunction by inhibition of cardiac progenitor cell proliferation and endogenous regeneration^30^. Mice overexpressing FrzA (Sfrp-1), an inhibitor of the Wnt signaling, had reduced scar size and cardiac rupture after MI, which are associated with reduced cell apoptosis in the scar region^31^. In mice with αMHC-Cre mediated β-catenin deletion, which made the cells non-responsive to Wnt stimulation, the scar size after MI was smaller which was associated with enhanced differentiation of a cardiac progenitor population (marked as αMHC^+^/GATA4^+^/Tbx5^+^, but cTnT^−^) into small cTnT^+^ cardiomyocytes in the infarcted region^32^.

In the present study we demonstrated for the first time that mice with cardiomyocyte-specific deletion of β-catenin have reduced susceptibility to ventricular tachyarrhythmias at 8 weeks after MI, which is associated with attenuated structural remodeling.

## Results

### β-catenin knockout reduces the susceptibility to ventricular tachycardias after myocardial infarction

**Figure 1:**
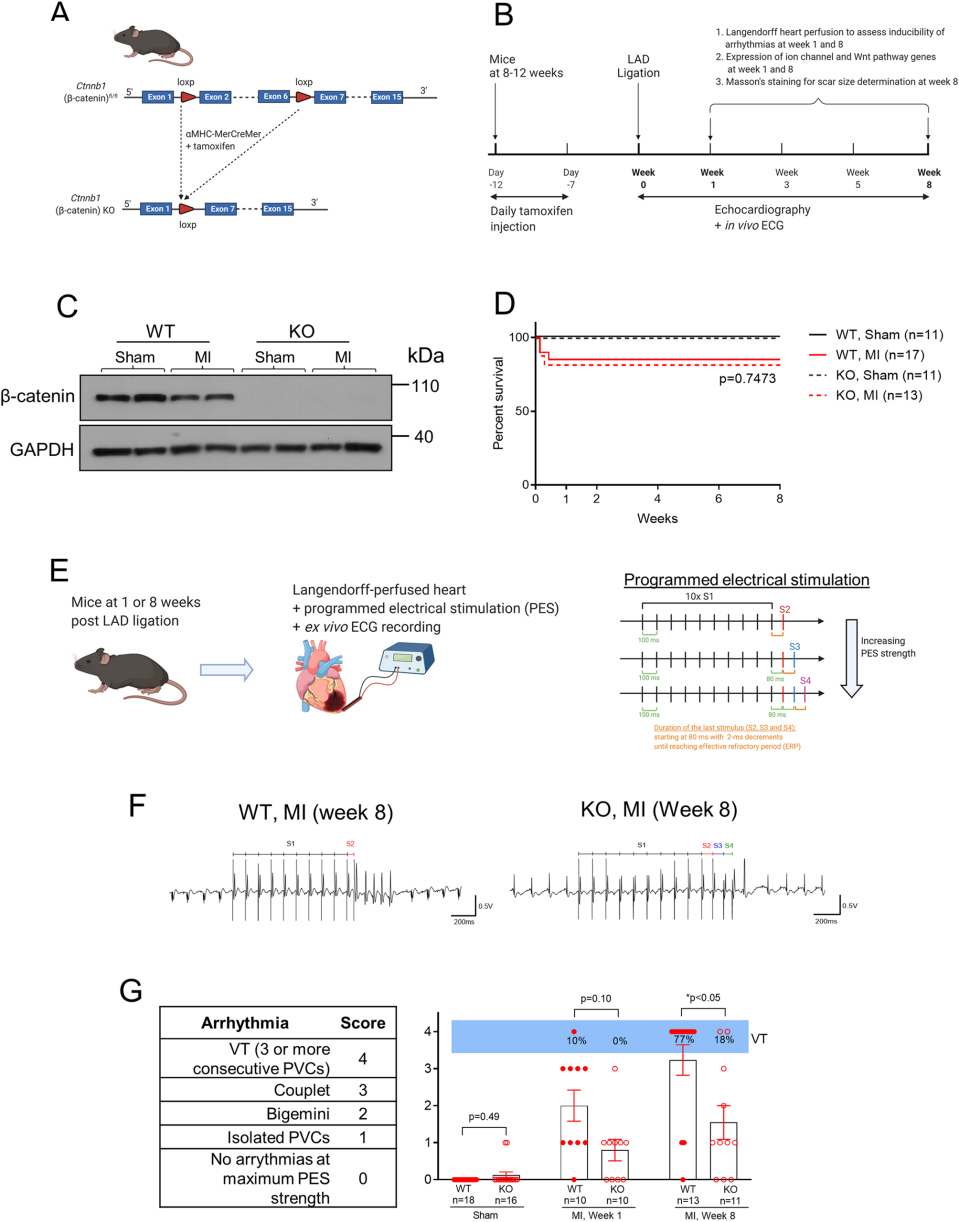
β-catenin knockout reduces the susceptibility to ventricular tachycardias after myocardial infarction. (**A)** To generate cardiomyocyte-specific β-catenin (*Ctnnb1*) knockout mice, *Ctnnb1*^flox/flox^ mice (with exons 2 to 6 floxed) were crossbred with αMHC-MerCreMer mice to obtain *Ctnnb1*^flox/flox^_;_αMHC-MerCreMer^+/−^ mice, which were then treated with tamoxifen for deletion of exons 2–6 generating *Ctnnb1* null allele due to the loss of the translational start site. Littermate *Ctnnb1*^flox/flox^_;_αMHC-MerCreMer^−/−^ mice were used as control wild-type mice. **(B)** Experimental design. (**C)** Representative western blot for confirmation of reduced total β-catenin in both sham and LAD-ligated (MI) KO hearts. Protein samples were prepared from left ventricular free wall of sham mice and from the border zone in mice at 8-weeks post-MI (myocardial infarction). GAPDH was used as a loading control. The original uncropped gel images are included in Supplementary Fig. 1. (**D)** Survival curve showing no difference in survival between WT and KO mice after LAD ligation (*p* = 0.7473, analyzed by Log-rank (Mantel-Cox) test). Only the mice in the long-term (8-weeks after MI) study were included in this survival analysis. All mortality occurred within the first week. (**E)** Protocols for evaluation of the inducibility of ventricular tachyarrhythmias (VT). *Left Panel:* Mouse hearts were isolated and Langendorff-perfused with Tyrode solution containing isoproterenol, ex vivo ECG were continuously measured by placing recording electrodes around the heart as we previously described^23,54^. Programmed electrical stimulation (PES) was applied using a MyoPacer (IonOptix) via a pair of platinum electrodes placed on the left ventricular apex of the heart. *Right Panel*: the standard stimulation protocol consisted of 10 stimuli at 100 ms intervals (S1, 5V) followed by one extra stimulus (S2) starting at an interval of 80 ms which was then reduced by 2 ms until the effective refractory period (ERP) was reached. If VT or VF was not induced, a second extra stimulus (S3) was added at 80 ms after S2. The S3 interval was then reduced by 2 ms until the ERP was reached. Finally, a third extra stimulus (S4) was added 80 ms after S3 and was then decreased by 2 ms until the ERP was reached. If a heart failed to develop a VT or VF with 3 extra stimuli, the heart was deemed non-inducible. (**F)** Representative ex vivo ECG (Lead II) showing PES-induced ventricular tachycardia (defined as 3 or more consecutive PVCs) in a WT heart (8-week after MI) when stimulated with one extra stimulus (S2), and only one single PVC in a KO heart (8-week after MI) when stimulated with three extra stimuluses (S4). (**G)** Summary of PES-induced PVCs and VTs in WT and KO hearts at 1 or 8 weeks after MI. An arrhythmia score was assigned to each heart according to the criteria shown in the left table. At 8 weeks after MI, VT was successfully induced in 77% of WT hearts but only in 18% of KO hearts. Data were analyzed by two-way ANOVA and Bonferroni *post-hoc* comparison.

Successful knockout of β-catenin after tamoxifen treatment was confirmed by western blot showing marked reductions of β-catenin protein in the left ventricle of *Ctnnb1*^flox/flox^_;_αMHC-MerCreMer^+/−^ (KO) mice as compared to *Ctnnb1*^flox/flox^_;_αMHC-MerCreMer^−/−^ (WT) mice (Fig. 1C). Post-MI survival curve showed that there was no difference (*p* = 0.75) in the death rate between WT and KO groups (Fig. 1D) with all the death (15% for WT; 19% for KO) occurred within the first week in both groups. The absence of animal deaths in the late stage after MI suggests no lethal arrhythmias in these mice. This reflects a known limitation of studying arrhythmias in mice—the low rates of spontaneous arrhythmias due to their fast heart rate, short action potential duration and smaller heart size, as compared to human and large animal models (e.g., monkeys and pigs) of heart disease.

To investigate the susceptibility of mice to ventricular arrhythmias, we used a protocol that we recently developed for rodent hearts^23,33^ that combined adrenergic stimulation (with isoproterenol) and progressive programmed electrical stimulation (PES) in isolated, Langendorff-perfused hearts (Fig. 1E), while ex vivo ECG was recorded by placing electrodes around the heart. With this protocol, ventricular tachycardias (VT, defined as three or more consecutive ectopic ventricular beats) were successfully induced in 77% (10/13) post-MI WT mice at week 8, but in none (0/18) of the sham-operated WT mice (Fig. 1F,G) validating our VT-inducing protocol. No difference was observed between male and female mice in VT inducibility (4/5 in male and 6/8 in female, *p* = 0.84) or arrhythmia score (Fig. 1G) (3.40 ± 0.60 in male and 3.13 ± 0.58 in female, *p* = 0.76). In contrast, VT was induced in only 18% (2/11) of the post-MI KO mice at week 8 indicating reduced susceptibility to ventricular arrhythmias (*p* < 0.05 vs. WT, Fig. 1G,). Interestingly, a significant portion of the KO mice at week 8 after MI (55% vs. 15% in WT) exhibited isolated or coupled premature ventricular contractions (PVCs) that did not develop into VT even when stimulated with the maximum PES strength (Fig. 1G). At week 1 after MI when the scar tissue was still immature, the majority of WT and KO hearts only exhibited isolated or coupled PVCs with VT induced in only one WT heart (Fig. 1G), suggesting a low inducibility of VT at this subacute phase.

### β-catenin knockout reduces the prolongation of QRS duration and QT interval after myocardial infarction

**Figure 2:**
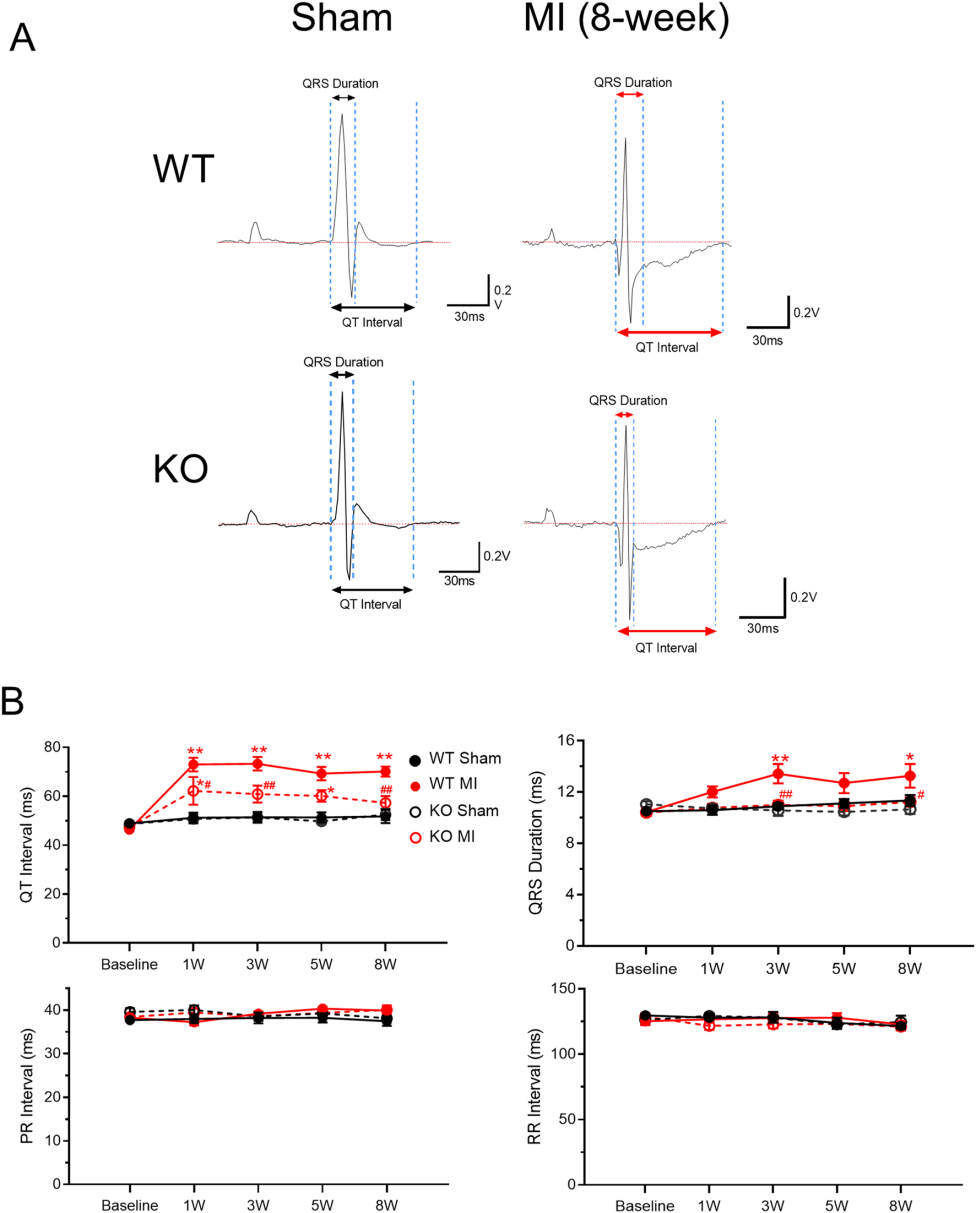
β-catenin knockout reduces the prolongation of QRS duration and QT interval after myocardial infarction. (**A)** Representative in vivo surface ECG traces (Lead II) in sham mice (left) and in mice at 8 weeks after MI (right). (**B)** Summary of in vivo ECG parameters, including QT Interval (top left), QRS duration (top right), PR Interval (bottom left), and RR Interval (bottom right). n = 10–13 per group. *p < 0.05, **p < 0.01, vs. corresponding sham groups; ^#^*p* < 0.05, ^##^*p* < 0.01, vs. WT MI group at the indicated timepoints. Data were analyzed by two-way ANOVA and Bonferroni *post-hoc* comparison.

Surface ECG recording in live animals showed no difference between sham-operated KO and WT mice in any of the parameters analyzed (QT interval, QRS duration, PR internal and RR internal, Fig. 2). WT mice that received LAD ligation (MI) surgery exhibited prolonged QT interval (starting at week 1) and increased QRS duration (starting at week 3), but showed no changes in PR or RR intervals, suggesting that MI led to left ventricular remodeling without significantly affecting the function of the central conduction system (sinoatrial node and atrioventricular node), although it is not clear if the intrinsic heart rate (i.e. after blocking the effects of autonomic nervous system) is altered. The MI-induced QT interval prolongation and QRS duration increases were attenuated in KO mice compared with WT mice (Fig. 2), which is consistent with reduced VT susceptibility in KO mice.

### β-catenin knockout attenuates chamber dilation and pump dysfunction after myocardial infarction

**Figure 3:**
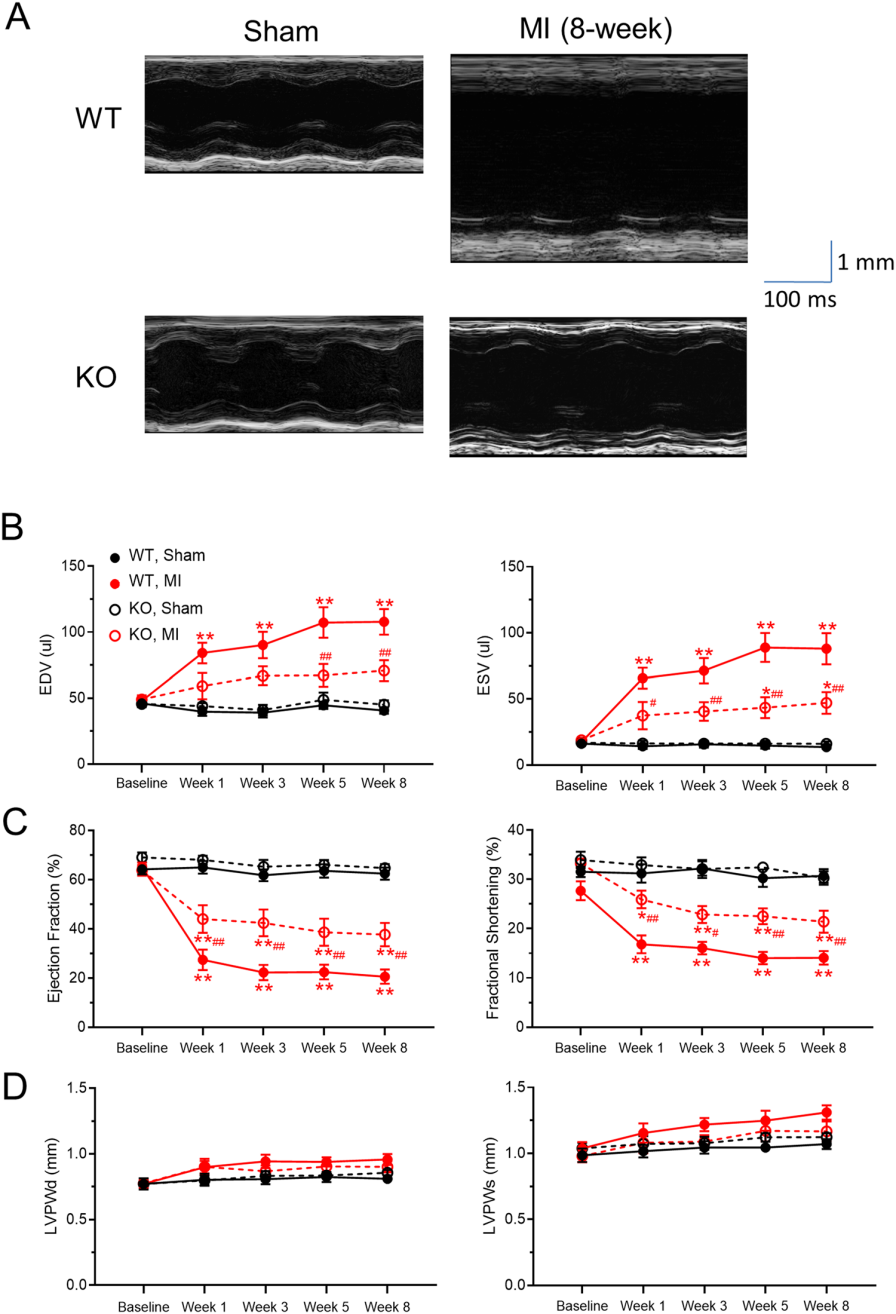
β-catenin knockout attenuates pump dysfunction and chamber dilation after myocardial infarction. (**A)** Representative short axis, M-mode echocardiographic recordings at the left ventricular mid-papillary level showing attenuation of cardiac function and dilation in β-catenin KO mice post-MI. Echocardiogram was recorded when heart rates were in the 400–500 bpm range. (**B) to (D)** Summary of echocardiographic parameters of mice up to 8 weeks after MI showing reduced left ventricular dilation in KO mice as measured by end diastolic volume (EDV, panel B, left) and end systolic volume (ESV, panel B, right), attenuation of cardiac systolic dysfunction in KO mice as measured by ejection fraction (panel C, left) and fractional shortening (panel C, right), and unchanged left ventricular posterior wall thickness at diastole (LVPWd, panel D, left) and at systole (LVPDs, panel D, right). n = 10–13 per group. *p < 0.05, **p < 0.01, vs. corresponding sham groups; ^#^*p* < 0.05, ^##^*p* < 0.01, vs. WT MI group at the indicated timepoints. Data were analyzed by two-way ANOVA and Bonferroni *post-hoc* comparison.

Echocardiography (Fig. 3A) showed no difference between sham-operated KO and WT mice in LV chamber dimension (end diastolic volume, EDV, Fig. 3B) or pump function (ejection fraction, EF, Fig. 3C). Post-MI WT mice exhibited marked LV dilation (EDV = 118.0 ± 16.5 µl, n = 14 vs. 42.0 ± 2.5 µl, n = 9 in sham WT at week 8, *p* < 0.01, Fig. 3B) and pump dysfunction (EF = 21.6 ± 3.3%, n = 14 vs. 64.7 ± 1.5%, n = 9 in sham WT at week 8, *p* < 0.01, Fig. 3C), but LV posterior wall thickness (LVPWd) was not changed (*p* = 0.7488 by ANOVA, Fig. 3D). In post-MI KO mice, LV dilation (EDV = 77.8 ± 7.8 µl, n = 13 vs. 44.4 ± 3.1 µl, n = 10 in sham KO at 8-week, p = 0.064, Fig. 3B) and pump dysfunction (EF = 34.9 ± 4.3%, n = 13 vs. 65.3 ± 1.1%, n = 10 in sham KO at 8-week, p < 0.01, Fig. 3C) were attenuated (*p* < 0.01) compared with post-MI WT mice.

### β-catenin knockout reduces scar sizes after myocardial infarction

**Figure 4:**
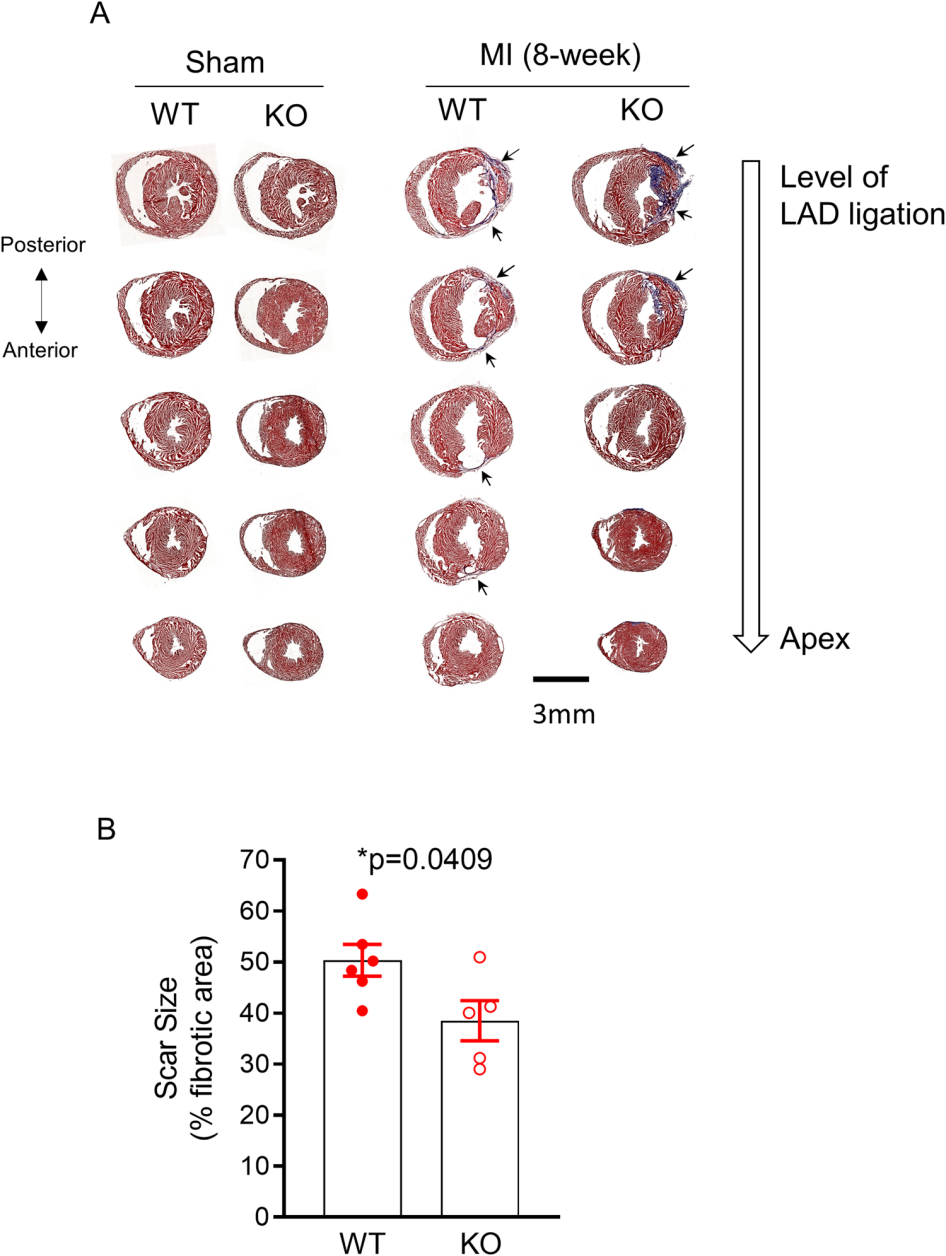
β-catenin knockout reduces scar sizes after myocardial infarction. (**A)** Representative images of Masson’s trichrome staining of mouse heart tissue sections at 5 different levels from LAD ligation site (top) to the apex (bottom) at 8 weeks after MI (right), as well as in sham-operated hearts (left). The scar size for each the 5 different levels was calculated as the percentage of fibrotic area (as indicated by the arrows) to the total area of the left ventricle section. The heart’s total scar size was then calculated as the mean of the scar sizes at the 5 levels and reported in panel B. (**B)** Summary of scar size showing reduced scar size in KO hearts (n = 5) compared to WT hearts (n = 6). Statistical analysis was performed with a two-tailed unpaired t-test.

Masson’s trichrome staining of sham-operated hearts did not show any difference in gross morphology between KO and WT mice (Fig. 4A). Consistent with observations in echocardiogram, post-MI WT hearts showed LV chamber dilation and wall thinning (Fig. 4A). The scar size, as determined by the % fibrotic area of the LV wall, was smaller in post-MI KO (Scar size 38.5 ± 3.9%, n = 5 vs. 50.3 ± 3.1%, n = 6 in post-MI WT at 8-week, *p* < 0.05, Fig. 4B).

### Upregulation of Wnt pathway genes in myocardium after myocardial infarction

**Figure 5:**
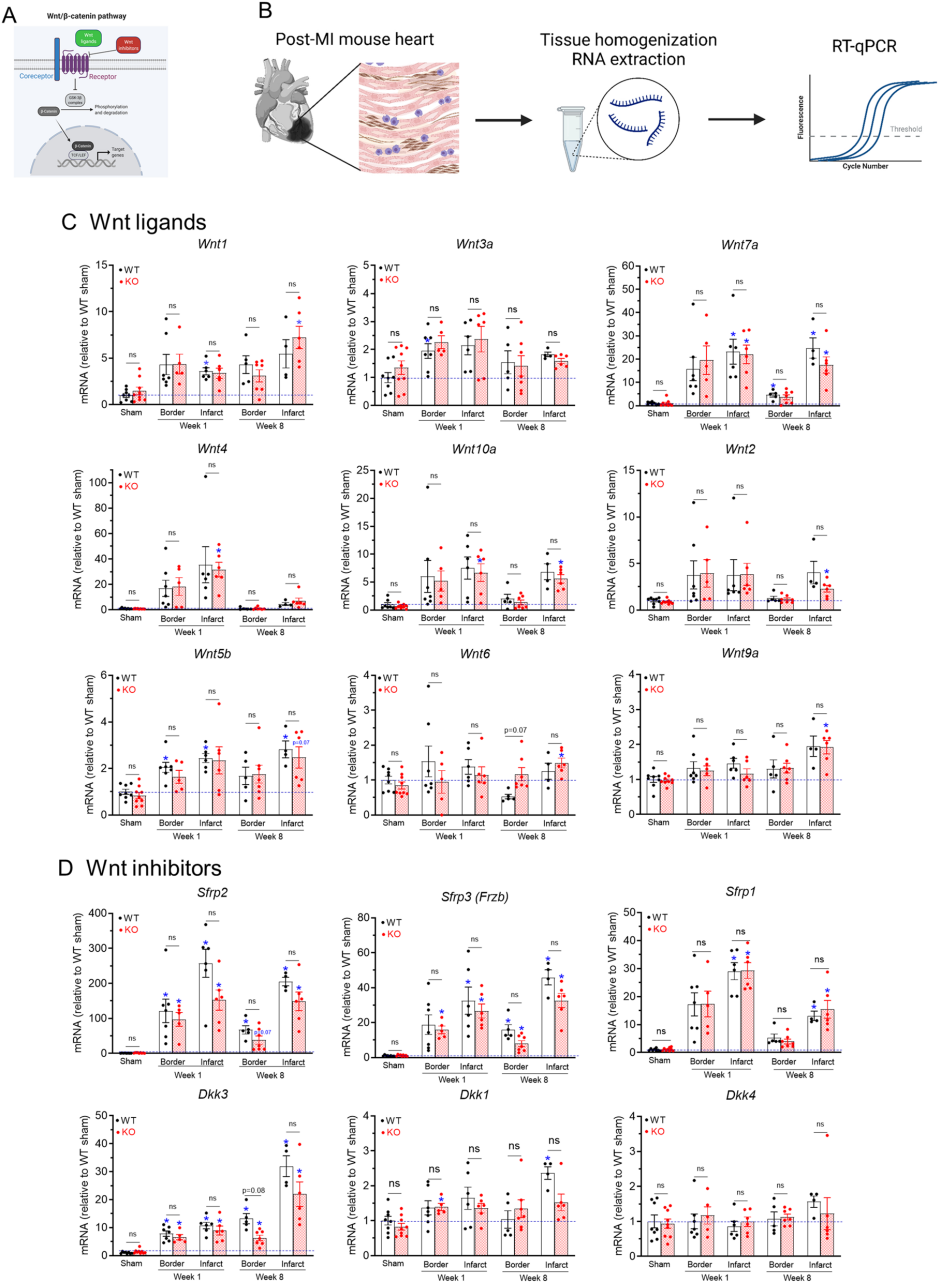
Upregulation of Wnt agonists and antagonists in myocardial tissues after myocardial infarction. (**A)** Diagram of the canonical Wnt/β-catenin signaling pathway, which is fine-tuned by the soluble Wnt ligands (green color) and Wnt inhibitors (red color) on the extracellular side of the plasma membrane. When a Wnt ligand binds to its plasma membrane receptor (purple color) and co-receptor (blue color), it leads to the inhibition of GSK-3β which is the key component of the β-catenin degradation complex; β-catenin will accumulate in the cytoplasm and then translocate into the nucleus where it, together with TCF/LEF, activate or inhibit the transcription of target genes. *Abbreviations*: GSK-3β, glycogen synthase kinase 3β; TCF/LEF, T-cell factor/lymphoid enhancer factor. (**B)** Detection of gene transcript levels from RNA samples extracted from heart tissues, which contained different cell types, such as cardiomyocytes (pink color), fibroblasts (brown color) and inflammatory cells (blue color). (**C**) and **(D)** qRT-PCR analyses of transcript levels of Wnt ligands (C) and Wnt inhibitors (D) in sham, 1-week post MI, and 8-week post MI infarct and border zone myocardium. n = 4–10 per group. *p < 0.05 vs. corresponding sham groups. “ns” means no significant difference between WT and KO groups. Data were analyzed by two-way ANOVA and Bonferroni *post-hoc* comparison.

**Figure 6:**
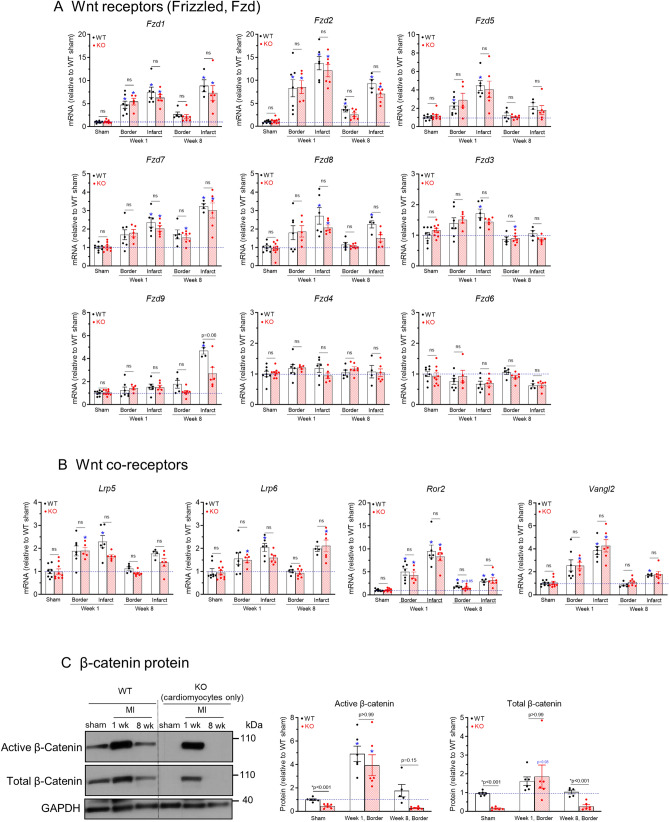
Upregulation of Wnt receptors and co-receptors in myocardial tissues after myocardial infarction. (**A)** and (**B)** qRT-PCR analyses of transcript levels of Wnt receptors (**A**) and Wnt co-receptors (**B**) in sham, 1-week post MI, and 8-week post MI infarct and border zone myocardium. n = 4–10 per group. (**C)** Western blot of border zone tissue (MI, n = 5–6) or left ventricular anterior free wall (sham, n = 5–6) with anti-non-phospho (active) β-catenin (Ser33/Ser37/Thr41) antibody and anti-total β-catenin antibody. GAPDH was used as a loading control. The original uncropped gel images are included in Supplementary Fig. 1. *p < 0.05 vs. corresponding sham groups. “ns” means no significant difference between WT and KO groups. Data were analyzed by two-way ANOVA and Bonferroni *post-hoc* comparison.

Wnt pathways are fine-tuned by the soluble Wnt ligands and Wnt inhibitors on the extracellular side of the plasma membrane, as well as the receptors and co-receptors on the plasma membrane (Fig. 5A). Our qPCR analyses of gene transcripts of the myocardial tissues, which contained both cardiomyocytes and other cell types such as fibroblasts (Fig. 5B), showed that a variety of Wnt ligands, inhibitors and receptors are upregulated in the border zone and infarct region of the mouse hearts after MI (Fig. 5 and 6).

#### *Wnt ligands* (Fig. 5C) 

*Wnt1*, a canonical Wnt ligand known to selectively activate the β-catenin pathway, was increased by 3–7 fold after MI (p < 0.05 in WT infarct at week 1 vs. WT sham; *p* < 0.05 in KO infarct at week 8 vs. KO sham) and *Wnt3a*, another canonical Wnt ligand, was increased by ~ 2 fold (*p* < 0.05 in WT border at week 1 vs. WT sham). *Wnt7*, a noncanonical Wnt ligand that selectively activate the β-catenin-independent pathways, was increased by ~ 20 fold in the infarct (*p* < 0.05 in WT infarct at both week 1 and 8 and border at week 8 vs. WT sham, *p* < 0.05 in KO infarct vs. KO sham). Six Wnt ligands (*Wnt4*, *Wnt10a, Wnt2, Wnt5b, Wnt6 and Wnt9a*) that have been reported to activate both β-catenin dependent and independent pathways or have less defined functions, were also increased after MI: *Wnt4* was increased by ~ 30 fold in infarct at week 1 (*p* < 0.01 in KO infarct vs. KO sham), *Wnt10* increased by ~ 5 fold in infarct (*p* < 0.05 in KO infarct at week 1 and 8 vs. KO sham), *Wnt2* showed a trend of increase in infarct at week 8 (*p* < 0.05 in KO infarct vs. KO sham), *Wnt5b* was increased by 2–3 fold (*p* < 0.01 in WT border and infarct at week 1, and *p* < 0.05 in WT infarct at week 8 vs. WT sham), *Wnt6* was increased by 49% in KO infarct at week 8 (*p* < 0.05 vs. KO sham), and *Wnt9a* was increased by 92% in KO infarct at week 8 (*p* < 0.05 vs. KO sham).

#### *Wnt inhibitors* (Fig. 5D)

Secreted frizzled-related proteins (Sfrp) are soluble Wnt inhibitors that prevent the binding between Wnt ligands and their plasma membrane receptors (frizzled)^34^. At week 1 after MI, *Sfrp2* showed the largest increase in both border and infarct (by 97–256 fold, *p* < 0.05 vs. WT sham or KO sham), *Sfrp3* also showed significant increases (by 16–32 fold, *p* < 0.05 vs. WT sham or KO sham) and *Sfrp1* was increased by ~ 30 fold in the infarct (*p* < 0.05 vs. WT sham or KO sham). At week 8, all three *Sfrp* levels have declined in border zone but remained high in the infarct region (*p* < 0.05 vs. WT sham or KO sham). Dickkopf-related proteins (Dkk) are another group of soluble Wnt inhibitors that selectively inhibit Wnt/β-catenin pathway by causing endocytosis of Wnt co-receptors (Lrp5/6) from the plasma membrane^35^. *Dkk3* was increased by 6–11 fold at week 1 (*p* < 0.05 vs. WT sham or KO sham) and was further increased to 22–32 fold in the infarct at week 8 (*p* < 0.05 vs. WT sham or KO sham). *Dkk1* was marginally increased by 1.3–2.0 fold (*p* < 0.05 in KO border at week 1 vs. KO sham, *p* < 0.05 in WT infarct at week 8 vs. WT sham), while *Dkk4* was not affected by MI (p = 0.801).

#### *Wnt receptors and co-receptors* (Fig. 6)

 Frizzled (Fzd) proteins are seven-transmembrane proteins that serve at the receptors for Wnt ligands on the plasma membrane (Fig. 5A). Among the 9 *Fzd* genes, the mostly affected by MI were *Fzd1*, *Fzd2* and *Fzd5* that were increased in border and infarct at week1 by 5–7 fold (*p* < 0.05 vs. shams), 8–13 fold (*p* < 0.05 vs. shams) and 2–4 fold (*p* < 0.05 vs. shams), respectively. But in the border zones at week 8 they all showed a significant decline. *Fzd7, Fzd8* and *Fzd3* in infarct were increased at week 1 (*p* < 0.05 vs. shams), and *Fzd7* and *Fzd8* in infarct remained high at week 8 (*p* < 0.05 vs. shams). *Fzd9* showed no changes at week 1 but was increased at week 8 in infarct (*p* < 0.05 in WT infarct vs. WT sham). The other two *Fzd* genes in the family (*Fzd4* and *Fzd6*) were not changed after MI (*p* > 0.20 and *p* > 0.22, respectively). Lipoprotein receptor-related protein 5 and 6 (Lrp5/6) are co-receptors required for Wnt ligand-induced activation of β-catenin pathway (Fig. 5A). *Lrp5* showed a 1.7–2.3 fold increase at week 1 (*p* < 0.05 in WT infarct and in KO border vs. shams). *Lrp6* also showed a 1.7–2.3 fold increase at week 1 (*p* < 0.05 in WT infarct and in KO border vs. shams), and a ~ twofold increase in infarct at week 8 (*p* < 0.05 in KO infarct vs. KO sham). But in the border zone at week 8 both *Lrp5* and *Lrp6* returned to near sham levels. *Ror2* and *Vangl2* are co-receptors required for non-canonical Wnt mediated activation of β-catenin-independent pathways. *Ror2* and *Vangl2* were increased in both border and infarct at week 1 by 4–9 fold and 2–4 fold (*p* < 0.05 vs. WT or KO shams), respectively, and they also showed a significant decline in border zone at week 8.

#### β-catenin protein

In cells not stimulated with Wnt/β-catenin pathway ligands, β-catenin in the cytoplasm is phosphorylated at Ser33/Ser37/Thr41 which labels them for ubiquitination and degradation^36^. The level of β-catenin that is not phosphorylated at Ser33/Ser37/Thr41 (i.e., active β-catenin) is an index of the Wnt/β-catenin pathway activation in the cells^37^. As shown in Fig. 6C, active β-catenin was increased by 4.9 fold in the border zone myocardium of WT hearts at 1 week after LAD ligation (*p* < 0.01 vs. WT sham), suggesting activation of the Wnt/β-catenin pathway in the myocardial tissue. An increase in active β-catenin at 1 week (3.9 fold, *p* < 0.05) was found in KO hearts in which only the cardiomyocytes were deleted of β-catenin. This suggests activation of the Wnt/β-catenin pathway at 1 week in non-cardiomyocytes (such as fibroblasts and inflammatory cells). In addition, the increased total β-catenin in KO hearts at 1 week also suggests an increased proportion of non-cardiomyocytes (e.g., by fibroblast proliferation and infiltration of inflammatory cells) in the border zone myocardium.

### Upregulation of Wnt pathway genes in ventricular myocytes after myocardial infarction

**Figure 7:**
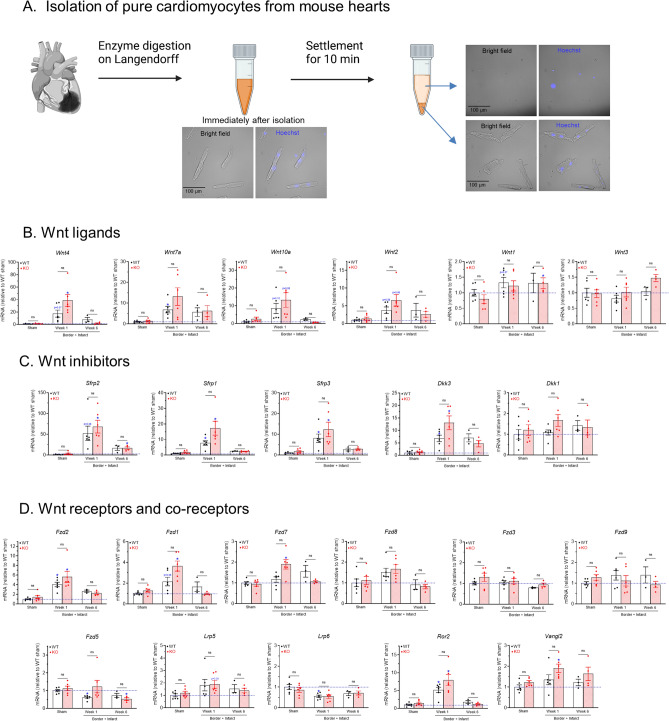
Upregulation of Wnt pathway genes in purified cardiomyocytes after myocardial infarction. (**A)** Isolation of pure cardiomyocytes from mouse hearts. Mouse hearts were digested with collagenase on a Langendorff perfusion system. The infarct and border region (or the left ventricular free wall in sham groups) were dissected, cut into small pieces, and gently pipetted to release single cells. The cell suspension contained both cardiomyocytes (rod-like cells) and other cell types (the Hoechst-positive small round cells), which can be separated by their different settlement speed: after a 10-min settlement, the cardiomyocytes were enriched in the pellet while the other cell types (non-cardiomyocytes) remained in the supernatant. The pellet containing pure cardiomyocytes was used for RNA extraction and qPCR analysis. The Hoechst-negative small particles are likely cell debris. (**B)** to **(D)** qRT-PCR analyses of transcript levels of Wnt ligands (**B**), Wnt inhibitors (**C**) and Wnt receptors and coreceptors genes (**D**) in pure cardiomyocytes isolated from sham, 1-week post MI, and 6-week post MI infarct and border zone. n = 3–6 per group. *p < 0.05 vs. corresponding sham groups. “ns” means no significant difference between WT and KO groups. Data were analyzed by two-way ANOVA and Bonferroni *post-hoc* comparison.

In order to investigate if Wnt pathway genes are altered in cardiomyocytes after MI, single cardiomyocytes were isolated from the infarct and border tissues (or from the left ventricular free wall in sham-operated groups) after collagenase digestion of the hearts (Fig. 7A). Cardiomyocytes were purified by removing non-cardiomyocyte cells after settlement for 10 min (Fig. 7A). qPCR analysis of pure cardiomyocytes showed that many of the Wnt pathway genes are also upregulated after MI (Fig. 7B to D).

#### *Wnt ligands* (Fig. 7B)

*Wnt4* was increased in cardiomyocytes by 17–38 fold at week 1 (*p* < 0.05 in KO vs. sham), which is similar to its increase in myocardial tissue (~ 30 fold). *Wnt10* showed a trend of 8–13 fold increase in cardiomyocytes at week 1 (*p* = 0.06 in KO vs. sham). *Wnt2* showed a trend of 4–6 fold increase in cardiomyocytes at week 1 (*p* = 0.06 in both WT and KO vs. shams). *Wnt1* was increased but to a lesser degree (by ~ 30%, *p* < 0.05) than in myocardial tissue (by 3–7 fold), while Wnt3a was not affected in cardiomyocytes (*p* > 0.25) after MI. *Wnt7* was increased by 6–13 fold (*p* < 0.05, WT week 1 vs. WT sham), which is less than its increase in myocardial tissue (20 fold).

#### Wnt inhibitors (Fig. 7C) 

*Sfrp2* was increased by 52–68 fold in cardiomyocytes at week 1 (*p* < 0.05 in KO vs. sham) and by 16 fold at week 6 (*p* < 0.05 in KO vs. sham), which were less than its increase in myocardial tissues (97–256 fold at week 1). *Sfrp1* and *Sfrp3*, which had ~ 30 fold increases in myocardial tissues at week 1, were increased in cardiomyocytes by 8–17 fold and 8–12 fold, respectively (*p* < 0.05 in both WT and KO vs. shams) but were not significantly affected at week 6. *Dkk3* was increased by 7–13 fold at week 1 (*p* < 0.05 in both WT and KO vs. sham) which is similar to its increase in myocardial tissues (6–11 fold), while *Dkk1* was not statistically affected (*p* > 0.09) after MI.

#### *Wnt receptors and co-receptors* (Fig. 7D)

*Fzd2*, which was increased by 8–13 fold in myocardial tissues at week 1, was increased in cardiomyocytes by 4–5 fold at week 1 (*p* < 0.05 in both WT and KO vs. shams). Similarly, *Fzd1*, which was increased by 5–7 fold in myocardial tissue, was increased in cardiomyocytes by 2–3 fold at week 1 (*p* < 0.05 in KO vs. sham). *Fzd7* was increased by 90% in KO at week 1 (*p* < 0.05 vs. sham). *Fzd3*, *Fzd8* and *Fzd9* were not statistically affected by MI (*p* > 0.57, *p* > 0.09 and *p* > 0.33, respectively). *Fzd5* was reduced by 40% in WT at week 1 (*p* < 0.01 vs. WT sham) and reduced by 54% in KO at week 6 (*p* < 0.05 vs. KO sham). *Lrp5* showed a trend of increase in cardiomyocytes at week 1 but was not statistically significant (*p* = 0.08 in KO vs. sham), while *Lrp6*, which had a ~ 2 fold increase in myocardial tissue, was reduced in cardiomyocytes by 45% at week 1 (*p* > 0.05 in WT vs. sham). *Ror2* was increased (by 5–8 fold, *p* < 0.01) to a similar level in myocardial tissue (4–9 fold) at week 1. *Vangl2*, which was increased by 2–4 fold in myocardial tissue, was increased in cardiomyocytes only by 54% in KO at week 1 (*p* < 0.05 vs. KO sham).

### Ion channel gene expressions after myocardial infarction

#### Ion channels genes for ventricular depolarizing currents (Fig. 8A)

 In the border zone at week 1, the cardiac Na^+^ channel gene transcript (*Scn5a,* encoding Na_v_1.5) was reduced in WT hearts (0.544 ± 0.07, n = 7 vs. WT sham 1.0 ± 0.07, n = 8, *p* < 0.01), but it was not reduced in β-catenin KO hearts (0.76 ± 0.15, n = 5 vs. KO sham 0.90 ± 0.04, n = 9, *p* > 0.99). Because it is known that Wnt/β-catenin signaling reduces *Scn5a* level in cardiomyocytes^22,23,25^ these observations suggest that the Wnt/β-catenin pathway likely plays a role in *Scn5a* downregulation in the border zone of post-MI hearts. In addition, the returning of *Scn5a* level to near sham level in WT border at week 8 (0.82 ± 0.05, n = 5 vs. sham 1.0 ± 0.07, n = 8, *p* = 0.38) also mirrored the reduced Wnt signaling at this time point. The significant reductions of *Scn5a* transcript in the infarct tissue at both week 1 and 8 are likely a result of the increased proportion of non-cardiomyocytes in this tissue. The L-type Ca^2+^ channel gene (*Cacna1c*, encoding Ca_v_1.2) was increased by 3.3 fold (*p* < 0.05) in both border and infarct tissues at week 1 as compared to sham groups. However, at week 8 *Cacna1c* in the border zone has returned to near sham levels. The high level of *Cacna1c* in infarct may reflect the expression of this channel in fibroblasts^38^ which are abundant in infarct tissues.

**Figure 8:**
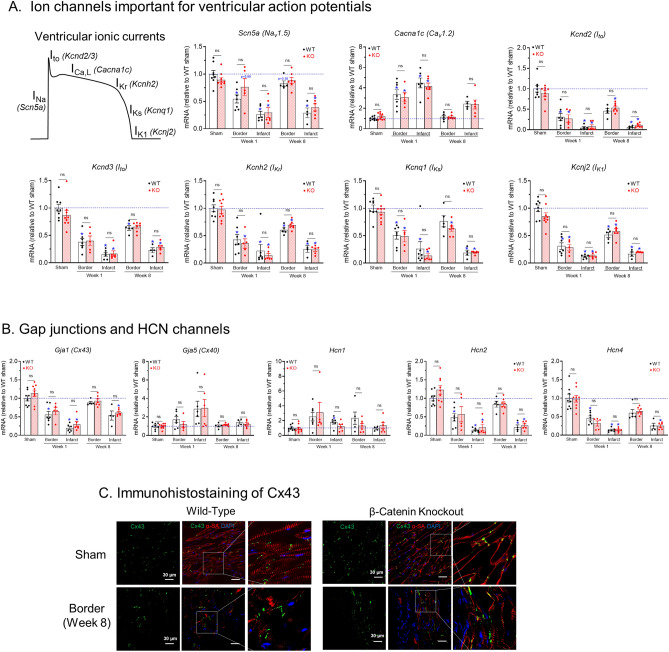
Ion channel gene expressions after myocardial infarction. (**A**) and **(B)** qRT-PCR analyses of transcript levels of ion channels involved in ventricular action potentials (**A**), and gap junctions and HCN channels (**B**) in sham, 1-week post MI, and 8-week post MI infarct and border zone myocardium. n = 4–10 per group. *p < 0.05 vs. corresponding sham groups. “ns” means no significant difference between WT and KO groups. Data were analyzed by two-way ANOVA and Bonferroni *post-hoc* comparison. *Abbreviations in panel A:* I_Na_, cardiac sodium current; I_to_, transient outward potassium current; I_Ca,L_, L-type calcium current, I_Kr_, rapidly activating delayed rectifier potassium current; I_Ks_, slowly activating delayed rectifier potassium current; I_K1_, inwardly rectifying potassium current. (**C)** Representative confocal images showing similar distribution patterns of Connexin 43 (Cx43, green) in WT and KO myocardium. In sham hearts (top panels) of both WT and KO groups, Cx43 is restricted to the cell–cell junctions. In the border zone of hearts at 8-week after MI (bottom panels), both WT and KO groups showed a more diffusive pattern for Cx43. Cells were co-stained with α-sarcomeric actinin (α-SA, red color, a marker of cardiomyocytes) and DAPI (blue).

#### Ion channels genes for ventricular repolarizing currents (Fig. 8A)

 All the major ventricular K^+^ channel genes, including I_to_ (*Kcnd2, Kcnd3*), I_Kr_ (*Kcnh2*), I_Ks_ (*Kcnq1*) and I_K1_(*Kcnj2*), were reduced (*p* < 0.05) in border and infarct tissues at both week 1 and 8 as compared to sham groups. Consistent with our previous findings that Wnt signaling does not affect I_K1_ or action potential repolarization^22^, no differences were seen between KO and WT groups in these K^+^ channel genes after MI.

#### Gap junctions and HCN channels (Fig. 8B)

 In the border zone, transcript of Cx43 (*Gja1*), the predominant gap junction isoform in ventricular myocardium, was reduced (*p* < 0.05) at week 1 but it returned to near sham levels at week 8 (Fig. 8B). However, immunostaining of Cx43 protein in border zones of both KO and WT hearts at week 8 showed that Cx43 is more diffusively distributed and is no longer restricted to cell–cell junction regions (intercalated disks) as seen in sham groups (Fig. 8C). Cx40 (*Gja5*), which is expressed in the Purkinje fibers, was not affected by MI. Among the genes of HCN channels that, if increased, will enhance myocyte automaticity, *Hcn1* was increased while *Hcn2* and *Hcn4* were reduced at week 1 but they returned to near sham levels at week 8 (Fig. 8B). No differences were seen between KO and WT hearts in these gene transcript levels.

## Discussion

Although Wnt/β-catenin signaling has been studied in animal models of ischemic heart disease, this is the first study to investigate the post-MI arrhythmogenesis using β-catenin knockout mice. The reduced susceptibility to VT in β-catenin KO mice after MI is likely due to attenuated structural remodeling. The low VT inducibility at week-1 in both WT and KO mice when the scar was immature and at week-8 in KO mice that had a smaller scar highlights the importance of structural remodeling in VT induction. Previous studies in patients have also demonstrated a positive relationship between scar sizes and the risk for VT^39^. In addition, large scar sizes are associated with a longer cycle length for monomorphic VT^40^, further supporting the reentry as the predominant mechanism for VT in post-MI hearts^41^.

The increased QRS duration, reflecting electrical dyssynchrony, is commonly found in heart failure patients and has been demonstrated to be an independent predictor of mortality in these patients^42,43^. Consistent with this, in the present study the increased QRS in post-MI WT hearts is associated with a greater VT inducibility. The increased QRS in these mice is likely secondary to the structural remodeling (scar and chamber dilation), as well as possible reductions in the conduction velocity of Purkinje fibers (arborization block) which is common in ischemic heart failure^44^. The QT interval and its dispersion are positively associated with the scar size in patients with MI and their increases are a risk factor for VT^45^. The attenuated increases in both QRS duration and QT intervals in β-catenin KO mice are consistent with their reduced VT inducibility and a smaller scar size.

The upregulation of Wnt pathway genes (such as *Wnt1* and *Wnt4*) in the myocardial tissue of post-MI hearts is consistent with previous studies^15,46^. In addition, the present study provided direct evidence for the upregulation of Wnt genes in the cardiomyocytes. The similar levels of *Wnt4* increase (~ 30 fold) in myocardial tissues and purified cardiomyocytes suggest that it is upregulated at the same degree in both cardiomyocytes and other cell types (such as fibroblasts, vascular cells and inflammatory cells). In contrast, other Wnt genes that are significantly increased in myocardial tissues are either upregulated at a lower level (such as *Wnt1* and *Wnt7*) or not increased (such as *Wnt3a*) in cardiomyocytes, suggesting differential levels of upregulation in these Wnt genes between cardiomyocytes and other cell types. This finding may be helpful for future designing of therapeutic strategies for cell type-specific interventions of the Wnt pathways in ischemic heart disease.

While previous studies have reported conflicting results regarding the role of β-catenin in post-MI structural remodeling, our observations that β-catenin KO mice had attenuated structural remodeling after MI are consistent with the study by Zelarayan et al*.*, which also found a smaller scar size in β-catenin KO mice at 4 weeks after MI (LAD ligation)^32^. The study by Zelarayan et al*.* also suggested that enhanced cardiac regeneration via activation of a cardiac progenitor population in the infarcted region may be a mechanism for the reduced scar in β-catenin KO hearts^32^. However, future studies are needed to investigate if other mechanisms, such as reduced cardiomyocyte deaths (in the form of necrosis, apoptosis, necroptosis and pyroptosis) or enhanced cardiomyocyte survival, are also involved. In addition to its role as the intracellular mediator of the Wnt/β-catenin signaling, β-catenin is also expressed in the cytoplasmic side of the plasma membrane in the cell–cell junction region, where it interacts with other membrane proteins such as N-cadherin^47,48^. Future studies are needed to investigate if the deletion of β-catenin in cardiomyocytes alters cell–cell interactions^46^ within the ischemic myocardium as a potential mechanism for the attenuated post-MI structural remodeling.

The reduced *Scn5a* transcript level in WT hearts, but not in KO hearts, at 1 week after MI is consistent with previous observations by us and others that Wnt/β-catenin signaling reduces *Scn5a* levels in cardiomyocytes^22–28^. Our finding that *Scn5a* transcript returned to near sham levels in WT hearts at 8 weeks after MI is also consistent with the reduced Wnt gene levels at this timepoint as compared to 1 week. In addition, the similar levels of K^+^ channel gene downregulations in both WT and KO hearts are consistent with our previous observation that Wnt/β-catenin signaling does not affect the resting membrane potential or the repolarization phase of action potentials in rat cardiomyocytes^22^. HCN channels have been shown to play a role in the arrhythmogenesis of dilated cardiomyopathy, and HCN2-overexpressing hearts have increased VT susceptibility^49^. In the present study, the selective upregulation of *Hcn1* gene is consistent with previous studies using a mouse model of cardiac hypertrophy, although *Hcn2* and *Hcn4* are more abundant than *Hcn1*^50^. One limitation of the present study is that ion channel genes were not analyzed in purified cardiomyocytes and fibroblasts. Of note, the cardiac fibroblasts are known to express voltage-gated K^+^ channels and transient receptor potential (TRP) channels^51^, and these cells can couple with cardiomyocytes in the myocardium to regulate electrical properties and arrhythmogenesis^51^. Future studies are needed to investigate the potential alterations in fibroblast ion channels and their contributions to the reduced arrhythmogenesis in β-catenin KO hearts after MI.

In summary, this study demonstrated that deletion of β-catenin reduces ventricular tachyarrhythmias in post-MI hearts primarily through attenuated structural remodeling. However, this study only examined the ischemic heart failure mouse model, and future studies are warranted to investigate the Wnt/β-catenin pathway activation in cardiomyocytes and its role in arrhythmogenesis in other types of heart failure, such as those induced by pressure overload or angiotensin II^17–19^.

## Methods

All procedures were approved by the institutional animal care committee at the University of Ottawa (Protocol #: HI-2602) and all methods were performed in accordance with the ARRIVE guidelines 2.0^52^. All methods were carried out in accordance with relevant guidelines and regulations.

### Mice

To generate cardiomyocyte-specific β-catenin (*Ctnnb1*) knockout mice, *Ctnnb1*^flox/flox^ mice (with exons 2 to 6 floxed, Jackson Lab, Stock No: 004152) were crossbred with αMHC-MerCreMer mice (Jackson Lab, Stock No: 005650) to obtain *Ctnnb1*^flox/flox^_;_αMHC-MerCreMer^+/−^ mice (Fig. 1A). Littermate *Ctnnb1*^flox/flox^_;_αMHC-MerCreMer^−/−^ mice were used as control wild-type mice. At the age of 8–12 weeks, all mice received daily subcutaneous injection of tamoxifen (20 mg/kg, Sigma, Catalogue No.:T5648, dissolved in sunflower seed oil at 10 mg/ml) for 5 consecutive days (Fig. 1B). At 7 days after the last tamoxifen injection, baseline surface ECG and echocardiogram were recorded before the experimental myocardial infarction surgery as described below. Both male and female mice were used to investigate any sex-specific effects. A total of 51 male mice (26 WT and 25 KO) and 65 female mice (34 WT and 31 KO) were used in this study.

### Experimental myocardial infarction

Myocardial infarction was induced in mice by permanent ligation of the left anterior descending (LAD) coronary artery as we previously described^53^. Mice were injected with buprenorphine (0.05 mg/kg; subcutaneous) 1 h prior to surgery and twice daily thereafter for 3 days. During the surgery, mice were incubated, anesthetized using 2% isoflurane and maintained under physiologic temperature. The heart was exposed after a left thoracotomy, the LAD was identified under a dissection microscope (Topcon Corporation, Model No.: OMS-75) and ligated at 0.3 mm distal to the atrioventricular junction using a Prolene 7.0 suture. Successful LAD ligation was confirmed by the pale color of the affected myocardium before chest closure with a Prolene 6.0 suture.

### Surface ECG

ECG with limb Lead I and II were recorded in mice at one day before and at week 1, 3, 5 and 8 after the LAD ligation using ADInstruments small animal ECG system (Powerlab 8/35 and Animal Bio Amp) and data were analyzed using LabChart 8.0. During ECG recording, mice were anesthetized with 1.5–2.0% isoflurane and body temperature was kept at 37 °C using a heating pad. Mouse ECG parameters were analyzed in LabChart 8 software (version 8.1.5, ADInstruments) software. In brief, automated averaging of 10 consecutive beats was done by the software. Three consecutive “averaged cycles” were analyzed, and these values were finally averaged. To manually correct the auto-detected intervals if they were incorrectly placed: the PR interval was measured from onset of the P wave to the onset of the QRS complex (in cases where the Q wave was not very visible, the onset of the R wave was used). The QRS duration was measured from onset of the QRS complex to the return to baseline voltage following the S wave (or if there was no return to baseline, the peak positive deflection following the S wave was used). The QT interval was measured from onset of the QRS complex to the return to baseline voltage following the T wave.

### Echocardiography

Echocardiography was recorded using a VEVO3100 system (VisualSonic Inc., Toronto) with the MS400 transducer (VisualSonic Inc., Toronto), and images were analyzed in VevoLab software (v.3.2.0 VisualSonic Inc., Toronto). During recording, animals were anaesthetized under 1.5–2% isoflurane with body temperature maintained at 37 °C using a heating pad. To minimize the influence of heart rate on pump function, echocardiogram was recorded when heart rates were in the 400–500 bpm range. M-mode tracing of the left ventricle (LV) was recorded in the short-axis view at the mid-papillary level. Three consecutive cardiac cycles, measured in VevoLab, were averaged to determine ejection fraction % (EF), fractional shortening % (FS), end-diastolic volume (EDV), end-systolic volume (ESV), left ventricular internal diameter (LVIDs/LVIDd), and posterior wall thickness. The EF was calculated using the following equation: (LVIDd^3^–LVIDs^3^)/LVIDd^3^ × 100. FS was calculated using the following equation: (LVIDd–LVIDs)/LVIDd × 100. B-mode tracing of the LV was recorded in the parasternal long-axis view and analyzed using the LV trace function in VevoLab to determine EF, FS, EDV, ESV, and LV volume.

### Programmed electrical stimulation (PES) in isolated mouse hearts

At 1 and 8 weeks after MI, mouse hearts were isolated and Langendorff-perfused with Tyrode solution (37 °C) containing 1 µM isoproterenol (Sigma, Catalogue No.: I6504), ex vivo ECG were continuously measured by placing recording electrodes around the heart as we previously described^23,54^ (Fig. 1E). PES was applied using a MyoPacer (IonOptix) via a pair of platinum electrodes placed on the left ventricular apex of the heart. The standard stimulation protocol (Fig. 1E) consisted of 10 stimuli at 100 ms intervals (S1, 5V) followed by one extra stimulus (S2) starting at an interval of 80 ms which was then reduced by 2 ms until the effective refractory period (ERP) was reached. If VT or VF was not induced, a second extra stimulus (S3) was added at 80 ms after S2. The S3 interval was then reduced by 2 ms until the ERP was reached. Finally, a third extra stimulus (S4) was added 80 ms after S3 and was then decreased by 2 ms until the ERP was reached. If a heart failed to develop a VT or VF with 3 extra stimuli, the heart was deemed non-inducible.

### Masson’s Trichrome staining

Mouse hearts were fixed by retrograde perfusion via the aorta with 4% paraformaldehyde (diluted in PBS) for 10 min at room temperature. The hearts were then incubated in increasing concentrations of sucrose dissolved in PBS (10% for overnight, 20% for 8 h, and 30% for overnight) on a horizontal rotator at 4 °C before being embedded in TissueTek OCT compound. Hearts were then cryosectioned at 10-µm slices using a Leica vibrating microtome (Model No.: CM3050S) at five different levels of the heart from the ligation site to the apex at 300-µm intervals. Ten consecutive slices were made for each level. Heart sections were then stained using a Masson’s Trichrome Staining kit (Sigma, Catalogue No.: HT15) according to the manufacturer’s instructions. Fibrosis areas were analyzed with ImageJ software using the Colour Deconvolution for Masson’s Trichrome Stain tool. The scar size for each the 5 different levels was calculated as the percentage of fibrotic area to the total area of the left ventricle section. The heart’s total scar size was then calculated as the mean of the scar sizes at the 5 levels and reported in Fig. 4.

### Immunohistostaining

Cryosectioned heart slices, as prepared above, were blocked and permeabilized in Dako protein block (Agilent, Catalogue No.: X0909) containing 0.1% (w/v) saponin (Sigma, Catalogue No.: 47036) at room temperature for 90 min. Slices were incubated with primary antibodies (see below) diluted in the same blocking solution at 4 °C for overnight. After washing with PBS, slices were incubated with secondary antibodies (see below) at room temperature for 1 h. After washing with PBS for three times, slices were mounted with ProLong gold antifade reagent containing DAPI (ThermoFisher, Catalogue No.: P36931). Primary antibodies used were polycloncal rabbit anti-Cx43 (1:200, Sigma, Catalogue No.: C6219), and monoclonal mouse anti-α-sarcomeric actinin (1:400, Sigma, Catalogue No.: A7811). Secondary antibodies used were anti-rabbit IgG (Alexa Fluor-488, 1:300) and anti-mouse IgG (Alexa Fluor-568, 1:300). Fluorescent images of the myocardium of left ventricular free wall of sham hearts and border zone of MI-hearts were taken with a ZEISS confocal microscope equipped with the Airyscan technique for high-resolution imaging (Zeiss Elyra S.1 LSM 880).

### Isolation of single ventricular myocytes

Single ventricular myocytes were isolated from adult mouse hearts according to a protocol we previously described^54–56^. Mouse hearts were isolated and perfused on a Langendorff system with a nominally calcium-free Tyrode solution (in mmol/L: 136 NaCl, 5.4 KCl, 0.5 Na_2_HPO_4_,10 HEPES, 1 MgCl_2_, and 10 glucose, pH = 7.40 with NaOH) at 37 °C for 10 min. Collagenase (1 mg/ml, type II, Worthington Biochemical Inc. Catalogue No.:LS004177) and protease (0.028 mg/ml, type XIV, Sigma, Catalogue No.: P5147) were added into the calcium-free Tyrode solution and the hearts were perfused for another 15–20 min. The left ventricular tissue containing the infarct and border zone (in MI hearts) or the left ventricular free wall (in sham-operated hearts) was dissected and cut into small pieces in KB solution (in mmol/L: 100 K-glutamate, 10 K-aspartate, 2.5 KCl, 10 KH_2_PO_4_, 2 MgSO_4_, 5 HEPES, 20 glucose, 20 taurine, 5 creatine, 0.5 EGTA, and 0.1% albumin, pH = 7.2 with NaOH). Single cardiomyocytes were released in KB solution by gentle pipetting. Cell suspension was kept on ice and allowed to settle for 10 min. A small aliquot (50 µL) of the cells were stained with Hoechst 33,342 (0.1 μg/ml, ThermoFisher, Catalogue No.: 62249) for 10 min and cell images were taken using a fluorescent microscope (Leica Microsystems, Model No.: DMi8) equipped with a DAPI filter cube and a 40 × objective. Cardiomyocytes settled to the bottom, while other cell types (such as fibroblasts, vascular endothelial and smooth muscle cells, and immune cells) remained in the supernatant (Fig. 7A). The cell pellet containing pure cardiomyocytes were used for RNA extraction and real-time PCR analysis.

### Real-time quantitative PCR (qPCR)

Total RNA was isolated from the border zone and infarct region of mouse hearts with Trizol reagent (ThermoFisher, Catalogue No.: 15596026) and digested with Turbo DNA-*free* kit (ThermoFisher, Catalogue No.: AM1907) to remove genomic DNA, according to manufacturer’s instruction. Total RNA from purified ventricular myocytes was extracted using a RNeasy Micro kit (Qiagen, Catalogue No.:74004) and genomic DNA were removed by on-column digestion with RNase-Free DNase kit (Qiagen, Catalogue No.:79254) according to instructions of the manufacturer. One µg RNA was used for cDNA synthesis with a High-Capacity cDNA Reverse Transcription Kit (ThermoFisher, Catalogue No.: 4368814). Real-time quantitative PCR was performed on a CFX Connect Real-Time PCR Detection System (Bio-Rad) using standard SYBR green method. Information of the qPCR primers was included in Table S1 in the Online Supplement. No reverse transcriptase (RT-) controls and no template controls (NTC) were included for all the primers to make sure that there were no genomic DNA contamination or primer dimer signals. Transcript level of target genes was normalized to the level of *Hprt1* mRNA in the same sample. Results were analyzed with the 2 ^-ΔΔ C(t)^ method.

### Western blotting

The border zone myocardial tissue of the post-LAD ligation hearts (week 1 and 8) or the left ventricular anterior free wall of sham-operated hearts were homogenized in T-PER Tissue Protein Extraction Reagent (ThermoFisher, 78510) containing Halt Protease Inhibitor Cocktail (ThermoFisher, 87786). Protein concentration was determined using Pierce Rapid Gold BCA Protein Assay Kit (ThermoFisher, A53226) and cell lysates (10 μg protein per lane) were run on a 4–12% SDS–polyacrylamide gel and transferred onto a PVDF membrane. The transferred membrane was incubated with a primary antibody (see below) overnight at 4 °C, followed by a 2-h incubation with a peroxidase-conjugated secondary antibody (1:2000, Cell Signaling, #7074). Primary antibodies used were rabbit anti-non-phospho (active) β-Catenin (Ser33/Ser37/Thr41) (1:1000, Cell signaling, #8814) and rabbit anti-total β-Catenin (1:1000, Cell signaling, #8480). Immunoreactivity was detected by chemiluminescence (Pierce™ ECL Western Blotting Substrate, ThermoFisher, 32209). Equal protein loading of the gels was assessed by re-probing the membrane with rabbit anti-GAPDH antibody (1:2000, Cell Signaling, #2118). Band densities were quantified using the “Gel Analyzer Protocol” function of ImageJ (https://imagej.nih.gov/ij/docs/menus/analyze.html#gels), and presented as values after normalization to GAPDH in the same samples. All the original uncropped gel images are included in Supplementary Fig. 1.

### Statistical analysis

Statistical analysis adhered to the Journal Guidelines. Data are expressed as mean ± SEM with *p* < 0.05 considered significant. Sample number indicates the number of biological replicates (each individual mouse is considered one sample). Differences between two means were evaluated by two-tailed Student’s *t*-test. Differences among multiple means were assessed by one-way or two-way analysis of variance (ANOVA). When significance was detected by ANOVA, differences among individual means were evaluated post hoc by Bonferroni’s test.

## Supplementary Information

Supplementary Information.
