# Supplementary material for: Cardiomyocyte-specific deletion of β-catenin protects mouse hearts from ventricular arrhythmias after myocardial infarction
Source: Sci Rep. 2021 Sep 6;11:17722. doi: 10.1038/s41598-021-97176-9 (PMC8421412; doi:10.1038/s41598-021-97176-9)

Online Supplement

Cardiomyocyte-specific deletion of β-catenin protects mouse hearts from ventricular arrhythmias after myocardial infarction

Jerry Wang[1,2], Ying Xia[1,2], Aizhu Lu[1,2], Hongwei Wang[1],

Darryl R. Davis[1,2], Peter Liu[1,2], Rob S. Beanlands[1] and Wenbin Liang[1,2,]

[1] University of Ottawa Heart Institute,

[2] Department of Cellular and Molecular Medicine, University of Ottawa, Ottawa, ON, Canada

Table S1. Primers for SYBR green qPCR analysis of mouse heart tissues

Gene	Forward sequence (5' - 3')	Reverse Sequence (5' - 3')	Amplicon Size (bp)	Target exon(s)
Wnt1	AGGGCGAACGACCGTGT	TCGGTTGCCGTAAAGGACG	169	3-4
Wnt2	GACCTGATGTAGACGCAAGGG	TGTAGCTCTCATGTACCACCAT	127	1-2
Wnt3a	CTACCCGATCTGGTGGTCCT	ACAGAGAATGGGCTGAGTGC	70	1-2
Wnt4	CGAGCAATTGGCTGTACCTGG	GTTTCTCGCACGTCTCCTCT	72	1-2
Wnt5b	GTTCCCTGAATGGCGGCTA	AGAGCTAGTGACCACCAGGA	179	1-2
Wnt6	TTCCAGTTCCGTTTCCGACG	CTGTCTCTCGGATGTCCTGC	85	2-3
Wnt7a	GAGATCAAGCAGAATGCCCG	TTCTCCTCCAGGATCTTCCGA	74	3-4
Wnt9a	CCTCGTGGGTGTGAAGGTGATA	CTTCATTGGTAGTGCTGCCC	179	3-4
Wnt10a	CATCTTCAGCCGAGGTTTTCG	AGCCTTCAGTTTACCCAGAGC	106	2-3
Fzd1	ACGAGGCTTACCAACAGCAA	CAGGCGATGGCTAGGATCAG	93	1
Fzd2	CCTCAAGGTGCCGTCCTATC	CAACACCGACCATGTGAGGA	151	1
Fzd3	GCAGATAGGTGGGCACAGTT	ATAGGGTGGAAGGGCTCCAT	150	2-3
Fzd4	TTCGGGGACGAGGAGGAG	ACCGAACAAAGGAAGAACTGC	194	1-2
Fzd5	TGTCGTTAAACTTTCCCAGCTCT	CTCCAAGGACAGAACTCTCGGA	113	1-2
Fzd6	CGGAGCCGCAGCAGTT	GGGACCTTTCCATCTTGCCA	186	1-2
Fzd7	TACGTGGGCCTGTCTAGTGT	CTTCTCTGTCTTGGTGCCGT	156	1
Fzd8	CCGCCTAGAGAAGGAGGACT	GAGCGAGGTCACTTCCAACA	75	1
Fzd9	CGTCGAGGTGTTCTGGTCTC	GAAGAAACACAACGCGGACC	79	1
Lrp5	TGCTCCCACATCTGTATCGC	AGGTAGGAGGCTCACCACAA	106	17-18
Lrp6	CTGCTGAGAGCGGCCC	CCAAACACAAAGTCCACCGC	137	1-2
Vangl2	GGGAAACAGGCGAGTGGTCT	GCAGCTCCCCGCCACT	196	1-2
Ror2	CTTCCCACTCTGAAAGGCTACT	CTTCGTGGCTCTTGCACAAC	164	1-2
Dkk1	CTCTTGACAACTACCAGCCCT	AGCACATAGCGTGCCTCAT	162	1-2
Dkk3	CATAAGCGTGTCAAGTGGAGC	GGAGTCCAAGTGACCGTCG	145	1-2
Dkk4	ACAAAGCAGTAAGGGACAGGA	TCCCTCTCGTAGAACTGGCT	118	3-4
Sfrp1	GCAAGCGAGTTTGCACTGAG	CCGCTTCAGCTCCTTCTTCT	126	2-3
Sfrp2	GCCACAGAGGAAGCTCCCAAG	GACACGCCGTTCAGCTTGTAA	194	1-3
Frzb	GCCGTTGTGGAAGTGAAGGA	GCCTTCTACCAAGAGTAACCTGG	174	4-5
Cacna1c	GACGTGCTGTACTGGGTCAA	CCCGCTAAGAACACCGAGAA	120	7-8
Gja1	CAGGTCTGAGAGCCCGAAC	TGTCTGGGCACCTCTCTTTCAC	111	1-2
Gja5	TGAGCTCTAAACGTGGAAGGC	CCGATGACTGTGGAGTGCTT	114	2-3
Kcnd2	CTCCACTATCCAACAGCCGA	TGCTGTGGTCACGTAAGGTT	89	5-6
Kcnd3	GCACACTCCACTACCACCTC	CAGAGCCTGGTGCGTCTC	72	1
Kcnj2	ACTTGCTTCGGCTCATTCTCT	CCAGAGAACTTGTCCTGTTGCT	116	1-2
Kcnh2	GTTTGAGGGCCAGAGCCG	TTGCAGTAGATGACAGCGCA	72	1-2
Kcnq1	CAGGGCCGAGTCTACAACTT	AGAACAATGAGGAAGACGGTGA	83	1-2
Hcn1	TGCTGGCGTTATCACCAAGT	CAGCAGGCATATCTCTCCGA	76	6-7
Hcn2	TGAACACCGGTACCAAGGGA	GAAGTTCACAATCTCCTCACGC	88	5-6
Hcn4	TGAGCCACTTCGAGAGGAGA	TGGAAGACCTCGAAACGCAA	129	5-6
Scn5a	TCCCAAGTTGTGTCTGGTGG	GTAACTGTCCTCGGGAGTCTCA	174	17-18

Supplementary Figure 1. Original uncropped gel images for Figure 1C and 6C.

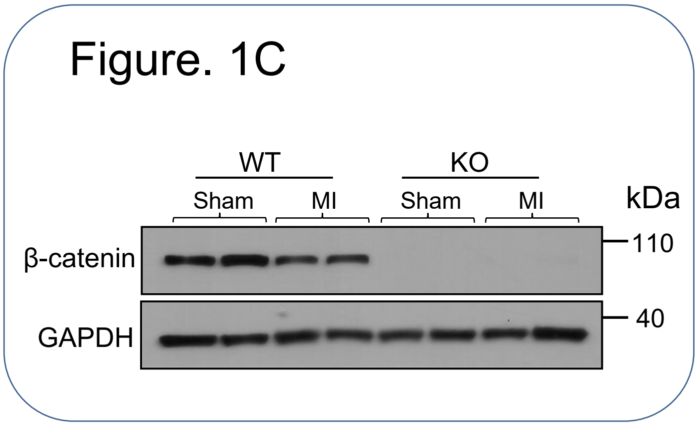

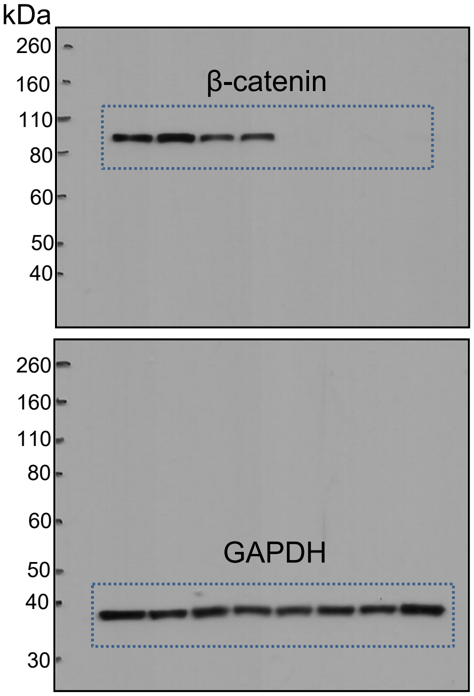

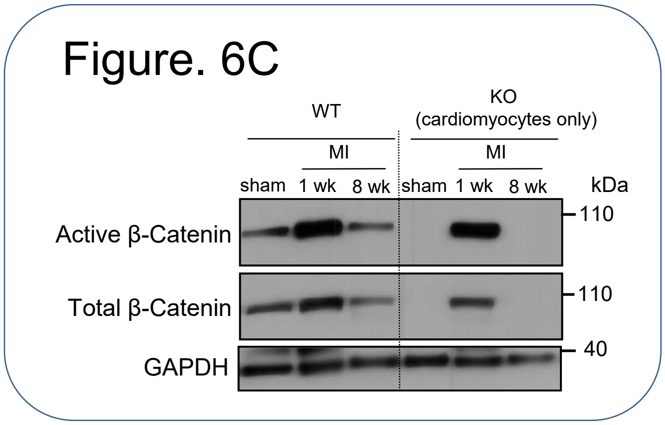

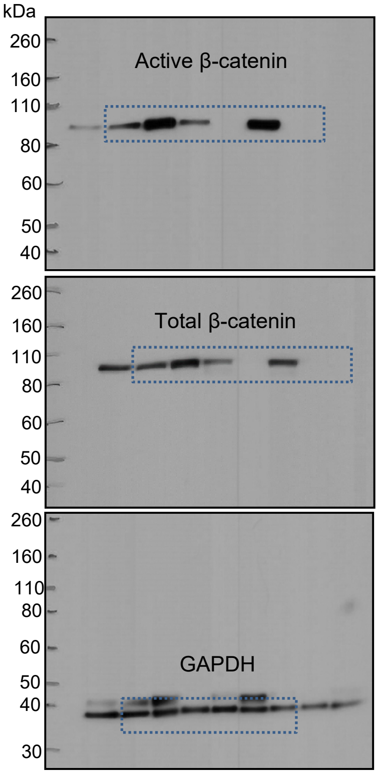

Supplement: Supplementary file 1 — Supplementary Information. [file 41598_2021_97176_MOESM1_ESM.pdf]
